# 365. Assessing Provider Utilization of COVID-19 Inflammatory Marker Trends in Hospitalized Patients and Implications in Optimizing Value-Based Care During a Pandemic

**DOI:** 10.1093/ofid/ofab466.566

**Published:** 2021-12-04

**Authors:** Praveen Subramanian, Lucy Stun, Nathan Bahr, Lewis Satterwhite, Maharshi Bhakta, Wissam El Atrouni, Fred Plapp, Jessica Newman

**Affiliations:** 1 University of Kansas Medical Center, Kansas City, Kansas; 2 The University of Kansas Medical Center, Kansas City, Kansas

## Abstract

**Background:**

Numerous inflammatory markers may serve a role in prognostication of patients hospitalized with COVID-19. Early in the pandemic, our health system created an admission order set which included daily d-dimer, c-reactive protein (CRP), lactate dehydrogenase (LDH), and ferritin. Given more available outcomes data, limiting standing order of studies that do not affect daily management could result in significant cost savings to the health system without adverse patient outcomes. The purpose of this study was to determine ordering and utilization patterns of inflammatory markers by physicians caring for patients hospitalized with COVID-19 infections.

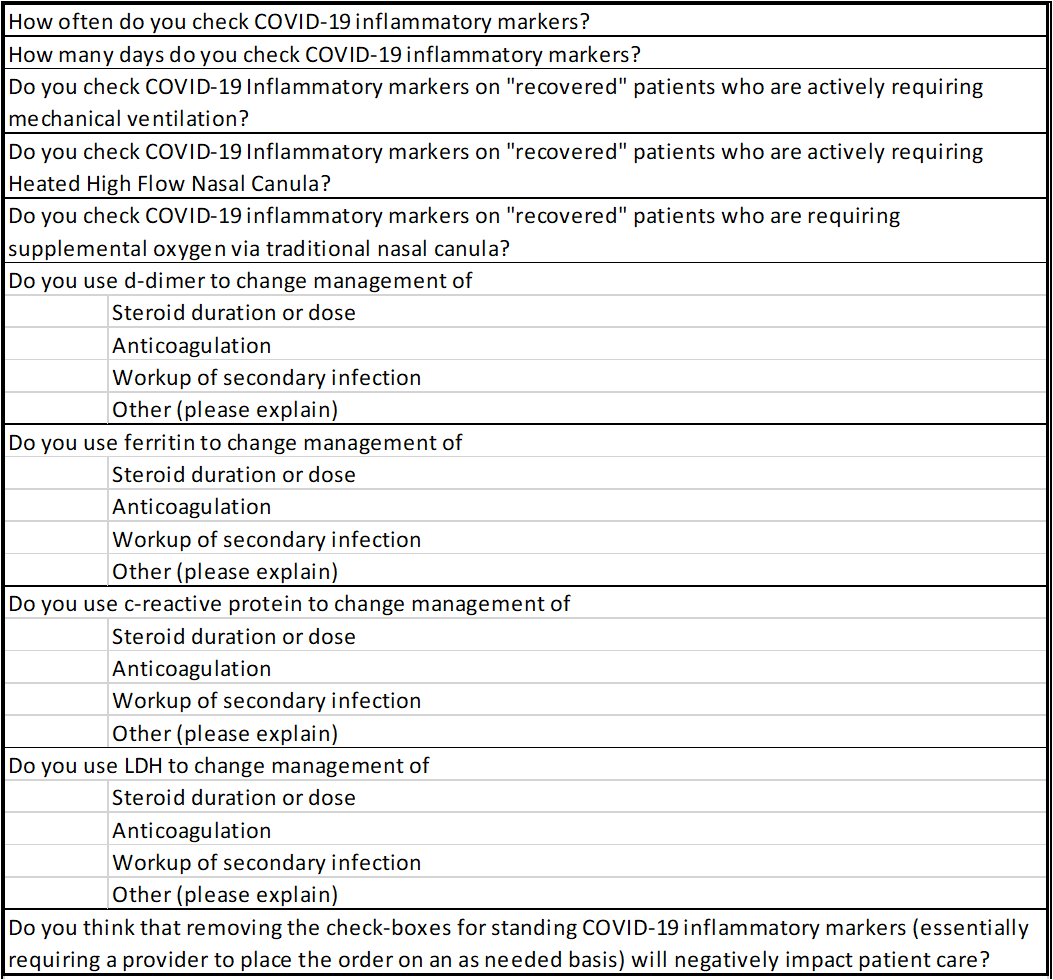

**Methods:**

An anonymous 10-question survey was distributed to 125 physicians (Infectious Diseases, Hospitalist, Pulmonary and Critical Care faculty). Responses were tallied and values greater than 50% were identified as the majority of the surveyed group.

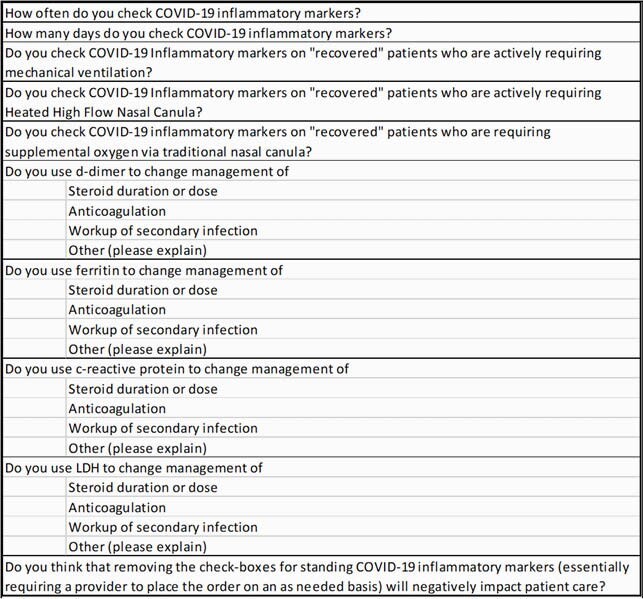

**Results:**

Of the 125 physicians surveyed, 77 (62%) responded. A total of 57.1% (44/77) of physicians reported ordering daily inflammatory markers for 3-10 days from admission. Another 31.2% (24/77) ordered markers until clinical improvement or hospital discharge. D-dimer was used for care decisions by 83.1% (64/77) of respondents; 93.8% (60/64) of those reported utilizing it in determining anticoagulation dose. CRP was used by 61% (47/77) of physicians to help identify a secondary infection or determine steroid dose or duration. LDH and ferritin were not used for management decisions by the majority of physicians. Inflammatory markers were not used routinely after isolation precautions had been discontinued, even when ongoing care required mechanical ventilation.

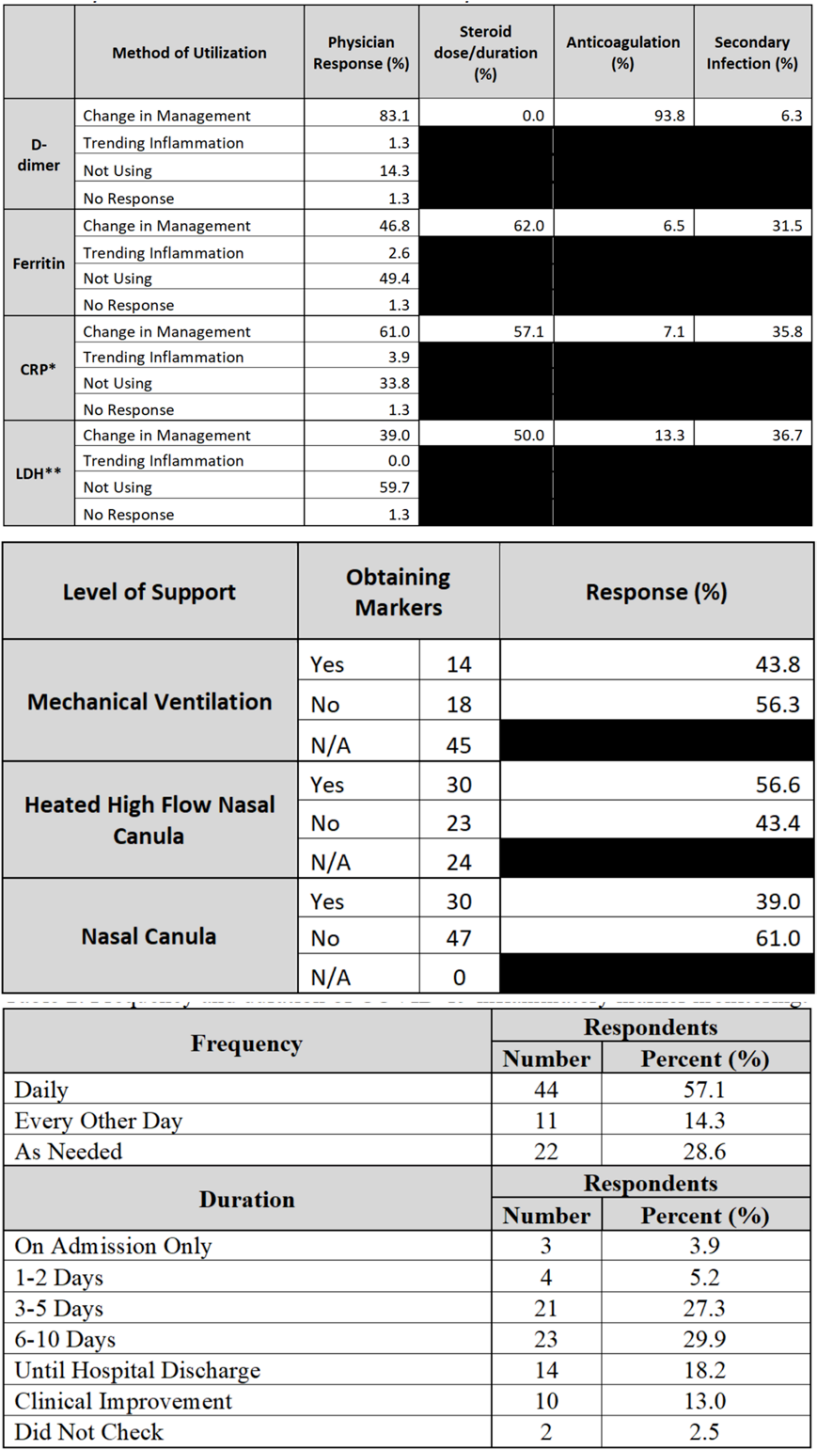

**Conclusion:**

Of the markers studied, both d-dimer and CRP were considered useful by most respondents. LDH and ferritin were used less frequently and were not considered as useful in guiding medical decision making. Discontinuation of standing daily LDH and ferritin orders is believed to have potential to result in cost savings to the health care system with no adverse patient outcomes.

**Disclosures:**

**All Authors**: No reported disclosures

